# Exploring Neurofilament Light Chain and Exosomes in the Genetic Forms of Frontotemporal Dementia

**DOI:** 10.3389/fnins.2022.758182

**Published:** 2022-01-25

**Authors:** Roberta Zanardini, Claudia Saraceno, Luisa Benussi, Rosanna Squitti, Roberta Ghidoni

**Affiliations:** Molecular Markers Laboratory, IRCCS Istituto Centro San Giovanni Di Dio Fatebenefratelli, Brescia, Italy

**Keywords:** neurofilament, NfL, exosomes, neurodegeneration, genetic frontotemporal dementia, presymptomatic carriers

## Abstract

Differential diagnosis of neurological disorders and their subtype classification are challenging without specific biomarkers. Genetic forms of these disorders, typified by an autosomal dominant family history, could offer a window to identify potential biomarkers by exploring the presymptomatic stages of the disease. Frontotemporal dementia (FTD) is the second cause of dementia with an age of onset < 65, and its most common mutations are in *GRN*, *C9orf72*, and *MAPT* genes. Several studies have demonstrated that the main proteins involved in FTD pathogenesis can be secreted in exosomes, a specific subtype of extracellular vesicles able to transfer biomolecules between cells avoiding cell-to-cell contact. Neurofilament light chain (NfL) levels in central nervous system have been advocated as biomarkers of axonal injury. NfL concentrations have been found increased in FTD and have been related to disease severity and prognosis. Little information on the relationship between NfL and exosomes in FTD has been collected, deriving mainly from traumatic brain injury. Current review deals with this matter in the attempt to provide an updated discussion of the role of NfL and exosomes as biomarkers of genetic forms of FTD.

## Introduction

Early diagnosis of dementia is challenging, that’s why there is an impelling need for specific biomarkers. Frontotemporal dementia (FTD) encompasses a heterogenous group of neurodegenerative disorders with a wide range of clinical, genetic, and neuropathological features (Bang et al., 2015). About one-third of FTD patients have an autosomal dominant family history (Rohrer et al., 2009), typified by mutations in three genes: granulin (*GRN*; Baker et al., 2006; Cruts et al., 2006), chromosome 9 open reading frame 72 (*C9orf72*) (DeJesus-Hernandez et al., 2011; Renton et al., 2011) and microtubule-associated protein tau (*MAPT*; Hutton et al., 1998). It has been demonstrated that several proteins involved in FTD pathogenesis can be secreted by cells in association with exosomes (Ghidoni et al., 2011; Benussi et al., 2016). Furthermore, mutations in *GRN* strongly reduce the number of released exosomes also altering their composition (Benussi et al., 2016). Exosomes are a specific subtype of extracellular vesicles (EVs) of 30–150 nm, originating in the endosomal/multivesicular body system and widely distributed in body fluids, including blood. Exosomes can carry a wide variety of DNA, RNA, proteins and lipids, allowing communication between cells avoiding cell-to-cell contact (Raposo and Stoorvogel, 2013; Rajendran et al., 2014). They have been reported as “Trojan horses” of toxic proteins (Ghidoni et al., 2008a), that’s why they may serve as novel biomarkers in neurodegenerative diseases (Rajendran et al., 2014; Longobardi et al., 2021). Exosomes have a lipid bilayer membrane and can cross the blood brain barrier bidirectionally, thus reflecting and tracking neuropathological changes (Chen et al., 2013; Lai et al., 2014). In this context, a number of studies have shown the potential of peripheral blood EVs enriched for neuronal origin (nEVs) to identify biomarkers in several neurological disorders (Mustapic et al., 2017). Moreover, encouraging studies have been published that illustrate how certain biomarkers of AD carried within circulating nEVs, can identify individuals with age-related cognitive decline at an early pre-clinical stage, when symptoms are milder than mild cognitive impairment (Eren et al., 2020). In the same line, significantly lower levels than controls of several excitatory synaptic proteins have been found in plasma nEVs in AD patients (Goetzl et al., 2018). In several neurological diseases, levels of neurofilament light chain (NfL) released from the Central Nervous System (CNS) have been demonstrated to be altered, mainly in the cerebrospinal fluid (CSF; Bridel et al., 2019) but also in serum (Mariotto et al., 2020). Concentrations of NfL, biomarker of axonal damage, are increased in serum of FTD patients and might be related to disease severity and prognosis (Meeter et al., 2016; Rohrer et al., 2016; van der Ende et al., 2019; Benussi et al., 2020). In the present review, the potential role of NfL and exosomes as promising biomarkers for FTD diagnosis are briefly explained in the context of the FTD forms typified by autosomal dominant mutations that allow investigations in the early or even in the presymptomatic stages of the disease.

## Genetic Bases of Frontotemporal Dementia

Frontotemporal dementia is an early-onset form of dementia, with a mean age of symptoms presentation before the age of 65 (Ratnavalli et al., 2002; Knopman et al., 2004). This early dementia is highly hereditary: 30–40% of FTD patients have a positive family history, (Rohrer et al., 2009; Wood et al., 2013; Fostinelli et al., 2018). In FTD families, null mutations in *GRN* leads to the production of a non-functional or no progranulin protein at all (Baker et al., 2006; Cruts et al., 2006; Ghidoni et al., 2008b; Finch et al., 2009; Sleegers et al., 2009). Mutations in *MAPT*, encoding for tau protein, typify FTD patients with tau-positive brain inclusions (Hutton et al., 1998; Poorkaj et al., 1998). Furthermore, an intronic expansion of a hexanucleotide repeat in *C9orf72* has been found in some families with an autosomal dominant inheritance form of FTD (DeJesus-Hernandez et al., 2011; Renton et al., 2011). Most forms of FTD, encompassing both genetic and sporadic FTD, are characterized by cell inclusion bodies composed of tau or transactive response DNA-binding protein of 43 kDa (TDP-43) (Greaves and Rohrer, 2019). TDP-43 cytoplasmatic inclusion can be found in the CNS of patient with FTD and/or amyotrophic lateral sclerosis (ALS) and could explain neuropathological overlap between these neurodegenerative diseases (Elman et al., 2008).

## The Role of Neurofilament Light Chain in the Genetic Forms of Frontotemporal Dementia

Neurofilaments are a family of neuronal cytoplasmic proteins divided into three subunits: heavy (NfH), medium (NfM) and light (NfL) chain. They are expressed primarily in neuronal axons where they provide structural support and stabilization of myelinated axons and interact with many proteins and organelles, including mitochondria (Petzold, 2005). NfL is the most abundant and soluble Nf subunit and can be released into blood and CSF in diverse neurological diseases reflecting neuroaxonal injury (Petzold, 2005; Lu et al., 2015; Mattsson et al., 2017; Khalil et al., 2018; Steinacker et al., 2018; Verde et al., 2019). NF gene mutations can cause multiple familial neurodegenerative disorders typified by NF aggregation and transport failure leading to further NF accumulations, including 14 NF-L gene mutations known to cause type 2E and 1F forms of Charcot–Marie–Tooth disease (Yuan et al., 2017). Recently NfL alterations have been also associated with FTD. NfL detection can provide some utility as a biomarker to differentiate specific FTD subtypes and, to support differential diagnosis of FTD from psychiatric disorders. To this regard, it has been recently reported an increase of serum NfL (sNfL) levels in behavioral-FTD but not in psychiatric disorders (Al Shweiki et al., 2019). Furthermore, low CSF level of NfL (cNfL) have been found in presymptomatic carriers of genetic FTD in contrast to high concentration in the symptomatic ones (Scherling et al., 2014; Meeter et al., 2016): in FTD *GRN*, *MAPT* or *C9orf72* mutation carriers, cNfL levels have reached a 8-fold higher increase in the affected patients than in presymptomatic carriers. On this basis, a role as a biomarker of disease severity and for prediction of the conversion to full dementia has been proposed for cNfL (Scherling et al., 2014; Meeter et al., 2016). Furthermore, it has been shown that sNfL levels were strongly associated with cNfL concentrations in the affected patients (Scherling et al., 2014; Meeter et al., 2016), and that NfL levels associated with disease severity, brain atrophy and patient survival (Meeter et al., 2016). In line with this evidence, van der Ende et al., 2019 showed normal sNfL levels in presymptomatic FTD carriers of *GRN*, *MAPT* or *C9orf72* mutations and a significant increase of sNfL concentrations after conversion to full dementia. The authors also described higher concentrations of sNfL in presymptomatic converters few years before the disease onset, pointing out to a potential role of sNfL as a prognostic biomarker of genetic FTD (van der Ende et al., 2019).

## The Role of Exosomes in the Genetic Form of Frontotemporal Dementia

Exosomes are biologically active entities, facilitating the intercellular communication and the transfer of biomolecules from one cell to another without direct cell-to-cell contact (Raposo and Stoorvogel, 2013; Rajendran et al., 2014). Alteration in intercellular communication in FTD patients with *GRN* mutation have been previously reported (Benussi et al., 2016). The study (Benussi et al., 2016) showed not only that progranulin was secreted in association with exosomes but also that levels of exosomal progranulin released by fibroblasts as well as the whole release of exosomes were reduced in mutations carrier patients. In brain, Wren et al., 2015, showed a significant alteration in intracellular vesicles trafficking with an accumulation of endosomes and exosomes and a reduction of lysosomes in FTD patients carrying N279K mutation in *MAPT.* These patients also showed an increase of exosomal proteins in frontal and temporal cortex (Wren et al., 2015). Moreover, it has been shown that both full length TDP-43 and TDP-43 C-terminal fragments were enriched in exosomes isolated from CSF in ALS-FTD patients. On this basis, approaches tackling the transmission of exosomes containing pathological TDP-43 could be a promising therapeutic strategy to halt or delay FTD-ALS progression (Ding et al., 2015). Based on these studies, exosomes and their cargo appear attractive biomarkers that could achieve a high diagnostic efficiency.

## Evidence on the Attractive Role of Neurofilament Light Chain in Exosomes

The interaction of NfL and exosomes in FTD has been preliminary explored in subjects with traumatic brain injury (TBI). A recent study focused on veterans evidenced that repetitive events of TBI were associated with elevated exosomal and plasma NfL: the years from the first TBI were associated with both plasma and exosomal NfL levels. However, the years since the last TBI positively correlated only with exosomal NfL (Guedes et al., 2020). Similarly, in the study from Peltz et al., 2020 on TBI, NfL in CNS-enriched exosomes isolated from plasma were associated with cognitive impairment, suggesting the utility of exosomal NfL as biomarker of cognitive loss. Conversely, the analysis of plasmatic NfL didn’t show any positive results (Peltz et al., 2020). Alongside a longitudinal study explored exosomal sNfL in patients with moderate-to-severe TBI in association with the free-circulating counterpart (Mondello et al., 2020). The authors found that sNfL levels were higher than their exosomal counterpart and that they positively correlated each other, likely part of a common disease process but pertaining to different pathways. Furthermore, exosomes enriched in sNfL were significantly higher in patients with diffuse TBI rather than in patients with focal lesions, supporting their potential utility in the prediction of neuronal damage (Mondello et al., 2020). In the same line, a study on HIV patients complaining neuropsychological impairment (Sun et al., 2017) showed that neuron-derived exosomes isolated from plasma had increased levels of NfL compared to exosomes from neuropsychologically normal subjects highlighting their usefulness in tracking the worsening of cognitive impairment.

## Conclusion

Even though not exhaustive, the present overview summarizes the most relevant evidence collected on the potential role of NfL and exosomes in the genetic form of FTD (Tables 1, 2). The latter can provide information on the presymptomatic stage of the disease, offering a good chance to identify early or prognostic biomarkers and the opportunity to deliver preventive therapeutic strategies in this ideal time to obtain the greatest possibility of success.

**TABLE 1:** Literature evidences of NfL alterations in genetic forms of FTD.

Mutated gene	Source	Presymptomatic mutation carriers	Symptomatic mutation carriers	References
*GRN, MAPT, C9orf72*	CSF	Levels of CSF NfL similar to CTRL	↑ CSF NfL vs. CTRL	Scherling et al., 2014
*GRN, MAPT, C9orf72*	CSF	Levels of CSF NfL similar to CTRL	↑ CSF NfL vs. CTRL and presymptomatic carriers (↑ CSF NfL in *GRN* patients compared to *MAPT* and *C9orf72* ones)	Meeter et al., 2016
*CHMP2B*	CSF	↑ CSF NfL vs. CTRL	↑ CSF NfL vs CTRL and presymptomatic carriers	Toft et al., 2020
*GRN, MAPT, C9orf72*	CSF	Levels of CSF NfL similar to CTRL	↑ CSF NfL vs CTRL and presymptomatic carriers	van der Ende et al., 2021
*GRN, MAPT, C9orf72*	SERUM	Levels of serum NfL similar to CTRL	↑ serum NfL vs CTRL and presymptomatic carriers (↑ serum NfL in *GRN* patients compared to *MAPT*)	Meeter et al., 2016
*GRN, MAPT, C9orf72*	SERUM	Levels of serum NfL similar to CTRL (↑ serum NfL in converters *vs* non-converter)	↑ serum NfL vs CTRL and presymptomatic carriers (↑ serum NfL in *GRN* patients compared to *MAPT* and *C9orf72*)	van der Ende et al., 2019
*CHMP2B*	SERUM	↑ serum NfL vs CTRL	↑ serum NfL vs CTRL and presymptomatic carriers	Toft et al., 2020
*GRN, MAPT, C9orf72*	SERUM	Levels of serum NfL similar to CTRL (↑ serum NfL in converters *vs* non-converter)	↑ serum NfL vs CTRL and presymptomatic carriers	Wilke et al., 2021
*GRN, MAPT, C9orf72*	SERUM	Levels of serum NfL similar to CTRL	↑ serum NfL vs. CTRL and presymptomatic carriers	van der Ende et al., 2021

**TABLE 2:** Literature evidences of exosomes alterations in genetic forms of FTD.

Mutated gene	Source	Mutation carrier patients	References
*GRN*	Human primary fibroblasts	↓ Exosomes *vs* CTRL	Benussi et al., 2016
*GRN*	Brain, Plasma	↑ Exosomes *vs* CTRL (only the symptomatic carriers)	Arrant et al., 2020
*MAPT*	iPSC-derived neural stem cells	↑ Exosomes vs. CTRL	Wren et al., 2015

Knowledge on the matter discussed is still at the beginning, and further investigation is needed to dissect the potential of this promising field of research and reveal whether the potential that emerged in the TBI study could also apply to genetic FTD. Exosomes represent an important subtype of EVs for the release and transfer of biomolecules among cells, without cells-to-cells contact. The study of the EV content, such as NfL in exosomes, from different tissues and fluids may provide information about the source of origin, reflecting the pathological changes. Moreover, it may predict the course of the disease and the prognosis for the patients, as well as establish a more reliable diagnosis. Since the first EVs description, ultracentrifugation has been the “gold standard” for EVs isolation. Nowadays, additional methodologies have been proposed for a more rapid and efficient EV isolation, such as several commercial kits, based on size exclusion.

Further prospective studies are greatly needed specifically to clarify the performance of exosomal biomarkers in genetic FTD diagnosis and prognosis.

## Author Contributions

RZ, CS, LB, RS, and RG gave their substantial contribution to conception and design of the manuscript and drafting the manuscript, revising it critically for important intellectual content. All authors have approved the manuscript in its present form for publication and agreed to be accountable for all aspects of the work in ensuring that questions related to the accuracy or integrity of any part of the work are appropriately investigated and resolved.

## Conflict of Interest

The authors declare that the research was conducted in the absence of any commercial or financial relationships that could be construed as a potential conflict of interest.

## Publisher’s Note

All claims expressed in this article are solely those of the authors and do not necessarily represent those of their affiliated organizations, or those of the publisher, the editors and the reviewers. Any product that may be evaluated in this article, or claim that may be made by its manufacturer, is not guaranteed or endorsed by the publisher.
